# Chitosan–Olive Oil Oleogels for Food Applications: Physicochemical and Functional Properties

**DOI:** 10.3390/foods14193332

**Published:** 2025-09-25

**Authors:** Álvaro Mosquera, Leticia Montes, Carlos A. Pena, Maria López-Pedrouso, Jorge Sineiro, Daniel Franco

**Affiliations:** 1 Department of Chemical Engineering, Universidade de Santiago de Compostela, rúa Lope Gómez de Marzoa, s/n., 15782 Santiago de Compostela, Spain; alvaro.mosquera.lorenzo@rai.usc.es (Á.M.); leticia.montes.martinez@usc.es (L.M.); jorge.sineiro@usc.es (J.S.); 2 CRETUS, Department of Chemical Engineering, Universidade de Santiago de Compostela, rúa de Constantino Candeira, 3, 15782 Santiago de Compostela, Spain; carlos.pena.puga@usc.es; 3 Department of Biochemistry and Molecular Biology, Facultade de Ciencias, Universidade de Santiago de Compostela, 27002 Lugo, Spain; mariadolores.lopez@usc.es

**Keywords:** rheological analysis, textural properties, color features, oxidative stability, lipid digestibility

## Abstract

The popularity of processed meats stems from modern demand for ready-to-eat foods, but their saturated and trans fats pose health concerns. Oleogel-based systems, which turn healthy oils into solid fat-like matrices, offer a promising alternative. This study characterized virgin olive oil oleogels structured with chitosan, assessing rheological, thermal, structural, and functional properties, examining how chitosan concentration (1–3%) and oil-to-water ratio (50–60) affect their performance. Rheological tests indicated a predominantly elastic behavior, suggesting the formation of stable gel networks, while a thermogravimetric analysis confirmed thermal stability of up to 237 °C, indicating suitability for moderate thermal processing. Texture analysis showed wider values for hardness (1.25–12.20 N) and color measurements indicated a homogeneous appearance across formulations with oleogels with high luminosity (L* > 50). The oleogels demonstrated high oil-binding capacity (>90%) and reduced oxidative degradation compared to bulk olive oil (peroxide values within regulatory limits for olive oils and TBARS values below 0.6 μmol malonaldehyde). In vitro digestion assays showed a slightly reduced lipid release with respect to pure olive oil, highlighting their potential for controlled lipid delivery and enhanced nutritional value. These findings support the potential of chitosan-based oleogels with virgin olive oil as stable and functional fat replacers in food applications.

## 1. Introduction

The high intakes of saturated fats (SFA) and trans fats (TFA)—commonly found in processed foods—have been consistently associated with adverse health outcomes. Evidence from large prospective cohort studies and meta-analyses show that elevated dietary intake of SFA is linked to increased risk of all-cause mortality, coronary heart disease (CHD), and type 2 diabetes. Similarly, TFA, particularly those of industrial origin, are strongly associated with higher rates of cardiovascular disease and premature mortality. Substituting just 5% of the total energy from SFA with polyunsaturated fats (PUFA) or plant-derived monounsaturated oils significantly reduces the risk of CHD and mortality, while replacing 2% of the total energy from TFA with plant monounsaturated fats (MUFA) has been shown to lower both CHD incidence and all-cause mortality [1]. Therefore, the replacement of SFA and TFA with healthier alternatives has become a growing priority in the meat industry, but in food products, which traditionally rely on animal fats for texture and flavor, this challenge is particularly significant. Oleogels, structured systems that convert liquid oils rich in unsaturated fatty acids into semi-solid forms, have emerged as a promising solution to mimic the functional properties of SFAs and TFAs while improving the nutritional profile of food products [2]. These oleogels can be designed to incorporate unsaturated fatty acids, offering a healthier lipid profile without compromising texture, flavor, or shelf-life. As such, the integration of oleogels into meat formulations aligns with consumer demands for healthier food options and regulatory trends aimed at reducing harmful fats in processed foods; hence, these systems are increasingly being explored in the food industry as alternatives to traditional solid fats [3].

Chitosan (CH), a biopolymer derived from the deacetylation of chitin, has emerged as a versatile material in biomedical, pharmaceutical, and food applications due to its biocompatibility, biodegradability, and non-toxic nature, along with solubility in acidic media, that facilitates its use in the aqueous phase of emulsions [4,5]. One of the novel applications of CH is its role as an organogelator in edible oils, because of the amino and hydroxyl functional groups, which facilitate hydrogen bonding and other intermolecular interactions necessary for gel formation. In addition, CH exhibits antioxidant activity, which can contribute to the preservation and stability of food products [6]. Indeed, the formation of oleogels using CH involves the self-assembly of polymer chains into a network that entraps the oil phase. This process is driven by non-covalent interactions such as hydrogen bonding, van der Waals forces, and hydrophobic interactions. But additionally, the use of aldehydes enhances the stability and three-dimensional structure of oleogels by forming Schiff bases through its reaction with chitosan’s amino groups [7,8]. Recent studies have highlighted the potential of chitosan as an oleogelator [5,9]. However, several factors influencing its performance, such as chitosan concentration [CH], temperature, oil-to-water ratio (O/W), and the presence of cogelators or emulsifiers, among others, have not yet been thoroughly investigated. One of the key factors influencing the Schiff reaction is the type of aldehyde employed. Previous studies have focused exclusively on vanillin; however, vanillin exhibits sensory notes that may be undesirable in certain applications and is also associated with a higher cost compared to other aldehydes. In contrast, the use of 4-hydroxybenzaldehyde offers clear advantages, as it is less expensive and its hydroxyl group promotes the formation of additional hydrogen bonds. Moreover, to the best of our knowledge, 4-hydroxybenzaldehyde has not yet been explored for this purpose.

Virgin olive oil is currently regarded as one of the healthiest vegetable oils due to its fatty acid profile (55–85% oleic acid and approximately 5–20% PUFA), as well as a minor fraction of bioactive compounds such as polyphenol derivatives, which exhibit strong antioxidant and anti-inflammatory properties. Moreover, numerous scientific studies have demonstrated that its consumption is associated with protective effects against various chronic diseases, including diabetes mellitus, osteoporosis, and neurodegenerative disorders [10]. The use of CH as an organogelator in olive oil offers a promising strategy to structure oils without compromising health benefits, aligning with global dietary recommendations. CH oleogels would improve the oxidative stability of olive oil by limiting its exposure to oxygen and light. These attributes make it an attractive candidate for developing functional food systems that not only mimic the texture of solid fats but also enhance the shelf-life of the final product. Certainly, these oleogels can be used to create spreads, dressings, bakery products, and processed meats that require a solid fat-like texture. Furthermore, the gel matrix can act as a carrier for apolar bioactive compounds, such as vitamins or polyphenols. Despite the promising potential of CH as an organogelator in olive oil, several challenges remain to be addressed, including the optimization of gelation conditions, assessment of stability under diverse storage environments, elucidation of interactions with other food ingredients, scalability of production, and regulatory approval for food-grade applications. As a first step, the study of key variables affecting the gelation process and their impact on the characteristics of the resulting oleogels deserve further investigation.

The objective of this study was to evaluate how chitosan concentration (1–3%) and the oil-to-water ratio (50/50 to 60/40) influence the rheological, physicochemical, thermal, textural, and functional properties of oleogels formulated with virgin olive oil, with particular attention to process-induced oxidation immediately after the drying step and in vitro digestibility.

## 2. Materials and Methods

### 2.1. Materials

Chitosan (85% degree deacetylation (DD), 96 mPa·s) and 4-hydroxybenzaldehyde (98%) were supplied by Sigma Aldrich Inc. (St. Lous, MO, USA). Virgin olive oil with an acidity percentage of less than 1% from Aceites Abril (Ourense, Spain) was acquired in a local retailer.

To ensure maximum accuracy in the amount of chitosan used for each emulsion, the water and impurity contents were determined. For water content, a gravimetric method was employed. Briefly, 0.5 g of chitosan was weighed on an analytical balance (A&D Weighing, HR-120, Tokyo, Japan), placed in a vacuum oven (Thermo Scientific, Heraeus Vacutherm VT 6025, Waltham, MA, USA), and dried at 70 °C under 100 mbar for 24 h until a constant weight was achieved (moisture content < 0.05%). Prior to weighing, chitosan samples were equilibrated in a desiccator, and the moisture percentage was calculated according to Equation (1).

(1) $$X\%=1-\frac{m_{f}}{m_{0}}\cdot 100$$

where $m_{0}$ is the initial weight and $m_{f}$ is the sample weight after drying.

For impurity determination, a chitosan solution was vacuum filtered using a Büchner–Kitasato system with filter paper (Whatman, 0.45 µm) that had been pre-dried and weighed. The retained solids on the filter paper were subsequently dried in a vacuum oven for 24 h at 70 °C and 100 mbar until achieving constant weight. The percentage of chitosan impurities was calculated using Equation (2).

(2) $$\%\;impurity=1-\frac{q_{f}}{q_{o}\cdot \left(1-X\%\right)}\cdot 100$$

where $q_{o}$ is the mass of chitosan used in the solution and $q_{f}$ is the final mass retained on the filter paper after drying. The chitosan powder used contained 5.94 ± 0.5% moisture and less than 0.01% impurities.

### 2.2. Preparation of Chitosan–Olive Oil Oleogels

#### 2.2.1. Preparation of Emulsions

The protocol used was based on previous works with slight modifications [7,11]. To produce emulsion, the continuous (aqueous) phase was composed of CH (1% *w*/*w* acetic acid) and 4-hydroxybenzaldehyde solution in ethanol 96%. After preparation, the pH was measured to confirm it was below 6. The amount of CH prepared in each solution varied depending on the desired final concentration in the oleogel; meanwhile, the amount of aldehyde used in the oleogel formulation was predetermined by the amount of CH added, maintaining in all cases a ratio of 1.3 mol of aldehyde per mol of CH. A wide range of CH concentration in the solutions was used according to the final concentration in the oleogel and the O/W ratio described in the Experimental Design and Statistical Analysis Section (Section 2.3.9).

To prepare the emulsions, an experimental setup consisting of a Rotaterm orbital shaker (Selecta, Barcelona, Spain), a high-performance dispersing Turrax (IKA, Ultra-Turrax T 25 basic, Staufen, Germany), and a 50 mL burette was used. The preparation began with a homogenization of CH, by placing the corresponding solution inside a beaker that was placed on the orbital shaker at 150 rpm, which was dispersed with the Ultra-Turrax at 9500 rpm for 30 s. This step is key to achieving proper emulsion. After 30 s, the olive oil was added to the CH solution using a burette for 70 s, maintaining the same stirring speed until the end of the process. Once all the oil had been added, the system was emulsified for 110 s till completion at 3 min, and finally, the aldehyde solution was added using a Pasteur pipette. Since the beginning of the oil addition, the total emulsion time was 8 min. The emulsion was left to rest for 24 h before the analysis and the subsequent drying stage. This time permitted the aldehyde to begin reacting with CH, despite low conversion in the presence of water.

#### 2.2.2. Drying Process

To obtain an oleogel from an emulsion, it is necessary to extract water so that the gelling agent traps only the oil within the molecular network. Additionally, this step is further enhanced by the reaction between the aldehyde and the CH. Emulsions (90 g) were dried in a convective dryer (ACS Angelantoni, Challenge 250, Massa Martana, Italy) following the methodology proposed by Lama et al. (2024) [9]. Briefly, emulsions were placed in Petri dishes in a uniform 12 mm thick layer. The thickness of the emulsion layer in the dish is a critical parameter during drying. The density of the emulsions was determined using a Ripette^®^ repeating pipette, due to the high viscosity of some emulsions. Drying conditions were set at 70 °C with 10% relative humidity and an air speed of 2 m/s. The total drying time was 3 h, which ended once the moisture content was less than 2%, according to Equation (3).

(3) $$X\;\left(\%\right)=\frac{m_{i}-m_{s}}{m_{s}}\cdot 100$$

(4) $$m_{s}=m_{i}\cdot (1-W)$$

where $m_{i}$ is the mass of the emulsion at a given time *i*, and $m_{s}$ is the theoretical dry mass of the emulsion *i*. This value can be obtained using Equation (4), where *W* is the mass fraction of volatile components in the emulsion (water, acetic acid, and ethanol). For this calculation, the amount of oil that could volatilize was disregarded, as it is practically negligible at the drying temperature (70 °C). After the desired drying level was reached, emulsions were removed from the dryer and wrapped in plastic film to prevent moisture absorption from the environment. Emulsions were left to rest for 24 h before the grinding stage, at room temperature, and in the absence of light, to avoid possible oil oxidation.

#### 2.2.3. Oleogel Homogenization

The grinding step involved a homogenization step using the Ultra-Turrax (IKA-Werke, Satufen, Germany). Grinding was carried out at 9500 rpm in thirty-second intervals, until the oleogel appeared uniform and free of large particulates and aggregates. Short intervals were used because longer durations could break the structure formed by the chitosan and the aldehyde due to the temperature increase caused by agitation and the shear stress applied. For conservation and subsequent analysis, oleogels were portioned into 5 g cubes using an ice cube tray lined with aluminum foil to facilitate their removal for subsequent analysis. To prevent oil oxidation, oleogels were covered with plastic film and stored in a refrigerator (~4 °C) for 24 h before any testing.

### 2.3. Analytical Methodology

#### 2.3.1. Microscopy Analysis of Emulsions

An optical microscope (Zeiss, Axioskop 40, Jena, Germany) equipped with a 20 MPixel digital camera (Kern Optics, ODC 841, Ebingen, Germany) was used to capture the images under brightfield illumination. Optical magnifications of 10× and 20× were used on an emulsion sample placed on a sample holder and covered by a coverslip. The parameters obtained were the number of retained drops per mm^2^ (ND), the average Feret diameter (µm), and the drop average area (µm^2^). The emulsions samples were studied twenty-four hours after preparation. To obtain a representative study for each emulsion, six samples were analyzed, with a total of four photographs per sample being shot. The images obtained were processed using ImageJ software v.1.54p (NIH, Bethesda, MA, USA), after applying FFT bandpass filtering and threshold adjustment.

#### 2.3.2. Rheological Analysis

A stress-controlled rheometer along with a RheoCompass™ software v 1.33 (Anton Paar, Physica MRC 301, Graz, Austria) was used for the analysis of the rheological behavior of the oleogels. Tests were performed using a parallel plate geometry (50 mm diameter) at a constant temperature of 25 °C using a Peltier heating system (±0.01 °C) and a thermostatic bath (Lauda, Ecoline Staredition E 100, Lauda-Konigshofen, Germany). The distance (gap) between the plates where the sample is placed was set to 1.5 mm for the oleogels. The linear viscoelastic region (LVR) was first determined via an amplitude sweep ranging from 0.01% to 10% strain at a fixed frequency of 1 Hz. Subsequently, a frequency sweep from 0.1 to 10 Hz was conducted at 0.1% strain to assess the viscoelastic properties of the samples.

#### 2.3.3. Thermogravimetric Analysis

The thermogravimetric analysis of the oleogels was performed in a TA Instruments TGA Q500 thermogravimetric analyzer (New Castle, PA, USA), using flow rates of 40 mL/min and 60 mL/min of nitrogen gas (Nippon Gases, 99.999%) as balance purge gas and sample purge gas, respectively. For each analysis, an open platinum pan containing 15–30 mg of sample was automatically introduced into the furnace chamber. The thermal program consisted of a heating ramp at 10 °C/min up to 600 °C. The software Universal Analysis 2000 v. 4.5A by TA Instruments was used to process the spectra.

#### 2.3.4. Textural Profile Analysis

The TPA test was carried out using a texture analyzer (Stable Micro System, TA.XTPlus, Surrey, UK) following Brito et al. (2022) [7] with slight modifications. Three 5 g oleogel cubes of 19.50 mm in diameter were used for each experimental point. A 25 mm diameter aluminum probe was used. Tests configured to achieve a compression distance on the sample corresponded to 50% of the total height with the following speeds: pre-test 2 mm/s, test 1 mm/s, and post-test 2 mm/s. The trigger force was set at 0.1 N and force-time curves were obtained. From these curves, the maximum hardness (N), cohesiveness (%), springiness (mm), and adhesiveness (N·s) were obtained.

#### 2.3.5. Color Parameters

Color oleogels were measured by a portable colorimeter (Konica Minolta LTD CR-400, Osaka, Japan). Before the measurements, the colorimeter was adjusted using a white ceramic tile (L* = 92.6000 a* = 0.3133, b* = 0.3195). Color parameters of CIELAB trichromatic coordinates of lightness, (L*); redness, (a*); yellowness, (b*) and values for hue angle (h*) and chroma (C*), were also calculated from Equations (5) and (6), respectively. Oleogels were measured 24 h after their elaboration, allowing 15 min for them to reach room temperature.

(5) $$h^{*}=\tan^{-1}\left(\frac{b^{*}}{a^{*}}\right)$$

(6) $$C^{*}=\sqrt{{a^{*}}^{2}+{b^{*}}^{2}}$$

#### 2.3.6. Oil-Binding Capacity

Oil-binding capacity (*OBC*) was determined according to the methodology described by Morales et al. (2023) [12], with minor modifications. Oleogels (1 g) were introduced in Eppendorf tubes previously weighed. The tubes were centrifuged (HWLAB, HW12, LinHai, China) at 13,500× *g* for 25 min at room temperature. After centrifugation was completed, the vials were left to rest in an inverted position for 10 min to ensure complete separation of the unretained oil. After this time, the supernatant oil (unretained) was removed with a Pasteur pipette from the sample and the remaining content in the vials was weighed again. The *OBC* (%) was calculated according to Equation (7).

(7) $$OBC(\%)=\left[1-\left(\frac{m_{1}-\left(m_{2}-m\right)}{m_{1}}\right)\right]\cdot 100$$

where *m* is the mass of the Eppendorf tube, *m*_1_ is the mass of the Eppendorf with the oleogel, and *m*_2_ is the mass of the Eppendorf after centrifugation and without any supernatant oil content.

#### 2.3.7. Primary and Secondary Oxidation Analysis

Firstly, oil from oleogels was extracted via centrifugation at 9576× *g* for 20 min. Primary oxidation was assessed by determining the peroxide value (PV), following AOCS methodology [13]. Briefly, 0.3–0.5 g of oil was dissolved in chloroform, followed by the sequential addition of glacial acetic acid, distilled water, and potassium iodide. The titration was carried out using a Titrator (Hanna HI901 Color, Woonsocket, RI, USA), and the PV was expressed in milliequivalents of oxygen per kilogram of oil (meq O_2_/kg).

Secondary oxidation was quantified using the thiobarbituric acid reactive substances (TBARS) assay, based on a modified protocol from Zhao et al. (2012) [14]. In this method, 0.5 g of each oleogel was mixed with a reagent solution containing 2% (*w*/*w*) hydrochloric acid, 0.375% (*w*/*w*) thiobarbituric acid, and 15% (*w*/*w*) trichloroacetic acid. The mixtures were incubated in a water bath at 95 °C for 15 min. After cooling to room temperature for 15 min, samples were filtered through a 0.45 µm membrane. The absorbance of the resulting supernatant was measured at 532 nm using a Genesys 10 UV spectrophotometer (Thermo Spectronic, Menlo Park, CA, USA). TBARS concentrations (μmol MDA/g oil) were calculated from the standard curve with 1,1,3,3-tetraethoxypropane and expressed as mg malonaldehyde/kg of oil.

#### 2.3.8. Lipid In Vitro Digestibility

The digestion procedure was based on in vitro INFOGEST protocol [15], with some modifications. Briefly, the initial amount of oleogel used was reduced to 1 g, based on the recommendation by Sabet et al. (2022) [16], who suggested that a lower quantity of oleogel improves the accuracy of in vitro digestion assays.

Two additional modifications were introduced: First, the NaHCO_3_ was replaced by NaCl at the same molar ratio to maintain ionic strength, thus preventing the undesired pH increase caused by the release of gaseous CO_2_. This modification was carried out following the procedure previously described by Mat et al. (2016) [17], who reported that this change improves pH stability during in vitro digestion. Secondly, the addition of bile salts was omitted, since only pancreatic lipase was used in the enzymatic mixture. The exclusion of bile salts was due to the absence of colipase; bile components may interfere with lipase activity and hinder lipid digestion according to Li et al. (2011) [18]. The percentage of free fatty acids (*FFA%*) released was calculated using the volume of NaOH consumed, according to Equation (8), assuming that lipase hydrolyzes two FFA per triglyceride molecule according to Li and Mcclements (2010) [19].

(8) $$FFA\%=\frac{\left(V_{NaOH}\cdot M_{NaOH}\cdot {MW}_{lipid}\right)}{2\cdot W_{lipid}}\cdot 100$$

where *V_NaOH_* is the volume (L) of the NaOH solution consumed to neutralize the FFA produced, *M_NaOH_* is the molarity (M) of the NaOH solution used, *MW_lipid_* is the average molecular mass of the triglycerides (879.67 g/mol), and *W_lipid_* is the total mass (g) of lipids present in the sample used for titration [20].

#### 2.3.9. Experimental Design and Statistical Analysis

The physicochemical properties of the oleogels were evaluated as a function of two independent variables: concentration of CH in the range of 1–3%, and the oil-to-water (O/W) ratio, ranging from 50/50 to 60/40. A three-level full factorial design (3^2^) was chosen to systematically evaluate the main effects and interactions of the studied factors. Table 1 describes the experimental points or systems elaborated, including four replicates at the center of the experimental domain for each dependent variable. The relationship between coded and uncoded variables is given by Equation (9).

(9) $$x_{i}=\frac{X_{i}-\bar{{X_{i}}_{i}}}{∆X_{i}}$$

where $X_{i}$ = real (uncoded) value of the factor, $\bar{X_{i}}$ = central point (midpoint) of the range studied, and ∆X_i_ = half − range (X_high_ − X_low_)/2).

The resulting data were analyzed using a first-order linear polynomial model with interaction terms (Equation (10)) applied to the coded variables (−1, 0, +1). The model fitting and statistical significance of coefficients were assessed via linear regression and ANOVA at a 95% confidence level using the IBM SPSS Statistics software.v.28

Y = b_0_ + b_1_·[CH] + b_2_·[O/W] + b_12_·[CH]·[O/W] (10)

where Y represents the dependent oleogel variable (estimated response); b_0_ is the intercept; b_1_ and b_2_ are the linear coefficients for the chitosan concentration and O/W ratio, respectively; and b_12_ is the coefficient for their interaction.

When both independent variables showed significant effects, Response Surface Methodology (RSM) was applied to explore their combined influence, using the Gnuplot software, v. 6.0 patchlevel 3. RSM plots were generated from the decoded variables to show the real experimental range. Each experimental point was measured in triplicate, and results were expressed as mean ± standard deviation. Duncan’s multiple range test (α < 0.05) was used to identify significant differences among means. Additionally, Pearson’s correlation coefficients were calculated to assess relationships between independent and dependent variables, using IBM SPSS.

## 3. Results and Discussion

### 3.1. Microscopical Features of Emulsions

**Figure 1:**
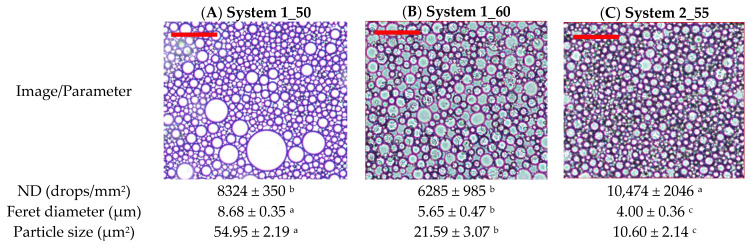
Optical microscopy images of experimental points: (**A**) system 1 (1_50), (**B**) system 3 (1_60), and (**C**) central design (2_55). Red bar = 100 microns. Different superscripts mean significant mean differences at 95% level confidence.

It should be noted that, for an oleogel system of a concentration of 3% CH and O/W ratio of 60/40 (Table 1), it was not possible to achieve an stable emulsion. During the emulsification process, a single stable homogeneous phase was never achieved because after completing the emulsification process, phase separation occurred, likely due to the depletion flocculation phenomena. Additionally, experimental point 2 (3% CH and O/W ratio of 50/50) produced hard and solid material, which could not be observed with optical microscopy. The ND of experimental points 1 (1_50) and 3 (1_60) were not significantly different (*p* < 0.05). In contrast, the biggest droplet size, measured as the Feret diameter, was obtained for system 1, being statistically different from system 2 and from samples from the central point (2_55). This result was due to the bimodal distribution of droplets observed in system 1 and not in system 3 (Figure 1A,B) where the fraction of small-sized droplets was more similar to the emulsions at the conditions of the central point of the design (Figure 1C).

The latter conditions rendered the most homogeneous emulsion with the smallest droplet size (4.00 ± 0.36 µm). This observation is according to the fact that the mass ratio is 55/45, which almost corresponds with the equality of volume (oil-to-water volumetric ratio 1:1) of the organic and aqueous phases, considering an oil density of 891 kg/m^3^. On the other hand, the average Feret diameter also decreased with the concentration of CH. This decrease was considerable between systems 1 and 3 (both at 1% CH) and the central system (2% CH). The different statistical groupings obtained for ND and the average Feret diameter values are due to emulsions from system 1 showing a very similar appearance to the central system for the fraction of smaller droplet size (a bimodal distribution not observed under other conditions), but containing a higher proportion of droplets with an area greater than 600 µm^2^ (Figure 1A). The oil in this type of droplet is likely to be released during the formation of the oleogel. The percentage of droplet area relative to the total area averaged was 3.02% for S1, 0.52% for S3, and 0.18% for the central system (Figure 1). These results will later support the oil retention values, which were lower under S1 conditions compared to the other systems. Both S1 and S3 have the same concentration of CH in the final oleogel (1%), differing only in the O/W ratio. This difference is reflected in the positive response function. Overall, an increase in the concentration of CH in the systems is associated with enhanced dispersion of the oil phase within the continuous phase, resulting in a higher ND and a reduction in their size.

These differences in droplet number and size could explain why a system with 3% CH and an O/W ratio of 60/40 could not be obtained. CH is generally considered a good stabilizer of O/W emulsions, based on two main parameters, such as the hydrophilic–lipophilic balance (HLB), which describes the type of emulsion formed (O/W or W/O) depending on the emulsifier used. The HLB ranges from 1 to 40, and for CH, it typically lies between 34 and 36.7, depending on its characteristics (mainly DD and MW). According to Klinkesorn (2013) [21], these values are adequate to ensure the formation of stable O/W instead of W/O. The second one is the emulsifying activity index (EAI) which defines the surface area of stabilized droplets (emulsion interface) per gram of the emulsifier. This index is useful to study differences between the emulsions produced with CH depending on concentration, MW, or DD [21].

Regarding DD, in the present study, a CH with a DD of 85% was used and stable O/W emulsions have been reported with similar DD values. Indeed, Del Blanco et al. (1999) [22] studied emulsions prepared with CH with a DD ranging from 73 to 95%, using sunflower oil as the dispersed phase at an O/W ratio of 20/80. All tested emulsions were stable, with no residual oil in the systems containing CH with DD values between 73 and 88%. However, above this DD threshold, residual oil droplets at the emulsion surface were reported. Therefore, the inability to form a stable emulsion in the abovementioned system may be attributed to the CH concentration and/or the O/W ratio. The depletion flocculation occurs when two droplets are adsorbed onto the same CH chain, forming a molecular bridge. Under these conditions, oil droplets are not separated by an independent CH layer and may interact because of the absence of sufficient repulsion. If this occurs across a large number of droplets, they could aggregate, and the emulsion could separate into phases. Such behavior has been observed in systems exceeding a certain critical CH concentration depending on MW and DD [21]. For example, in the system formulated with (3% CH, O/W of 50/50) which has the same final CH concentration as the system that failed, differing only in the O/W ratio (50/50 vs. 60/40)—a stable emulsion was obtained. This suggests that although the final CH concentration in the oleogels was the same (3%), the effective CH concentration in the precursor emulsion was higher in the system than that which could not be stabilized. The explanation lies in the fact that emulsions are less stable when smaller oil droplet sizes are formed, as this promotes flocculation in agreement with Klinkesorn (2013) [21] who reported that droplet size depended on the CH concentration in the emulsion; hence, higher CH concentrations lead to greater oil dispersion within the molecular network and, consequently, smaller droplets. In line with this, Payet and Terentjev (2008) [23] hypothesized that a higher [CH] in an emulsion leads to a decrease in interfacial tension between phases, since the non-deacetylated monomers adsorbed on the surface of the dispersed phase hinder phase contact. As a result, a larger interfacial area is required to achieve the same effect, thereby increasing droplet size. Despite our microscopy findings pointing to this fact, this statement is valid when the amount of oil added to the system remains constant; hence when the O/W ratio changes, a deeper understanding of how this second variable influences emulsion stability is required, and more studies should be conducted in this sense.

### 3.2. Rheological Analysis of Oleogels

**Figure 2:**
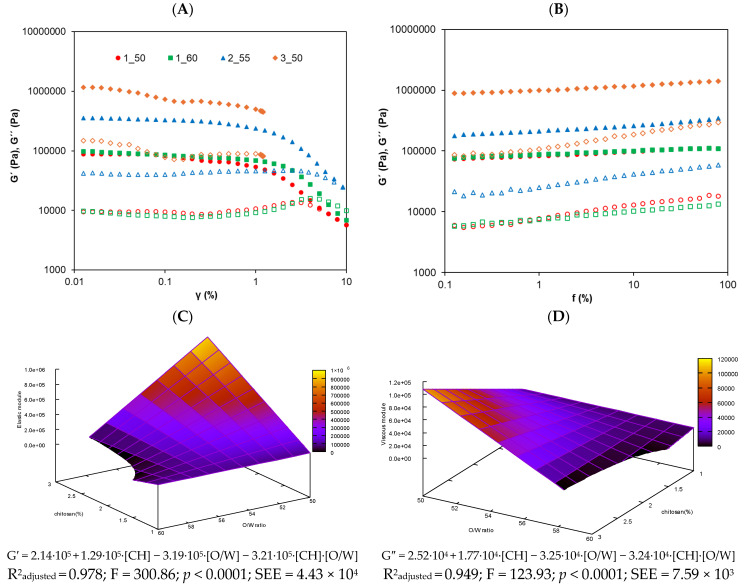
Rheological graphs for the studied oleogels: (**A**) strain sweep from 0.01% to 10% strain at 1 Hz and (**B**) frequency sweep from 0.1 to 100 Hz at 1% strain. G′ (elastic module) is represented with filled symbols and G″ (viscous module) with open symbols. (**C**,**D**) are the response surface for elastic and viscous module, respectively, of the olive oil oleogels.

Strain sweep tests were conducted to study the LVR. Figure 2A shows the strain sweep, where all oleogels exhibited the expected behavior, displaying a dominant elastic modulus (G′) over the viscous modulus (G″), which is consistent with previous findings reported by Zou et al. (2020) [24]. It was observed that the oleogels with the highest concentration of CH (3_50) exhibited fracturability upon exiting the plateau region, making it impossible to obtain reliable data beyond that point.

This brittleness could be attributed to the highly rigid and inflexible molecular structure formed by the strengthening of the molecular network due to the reaction between CH and 4-hydroxybenzaldehyde [11]. The critical strain, identified by a drop in G′, varied slightly among the oleogels. Based on these results, a strain of 0.01% was selected for the frequency sweep tests to ensure that all samples were analyzed within the LVR.

As shown in Figure 2B, G′ remained higher than G″ throughout the entire frequency range, confirming the elastic behavior of the studied systems. A slight frequency dependence of the moduli was observed, which is a typical characteristic of gels [25]. The 3_50 system stood out as the stiffest system, followed by 2_55, 1_60, and 1_50. These differences are in concordance with the composition of the oleogels, since the 3_50 oleogel system contains the highest concentration of CH (3%), promoting a more rigid and robust network. The 2_55 sample with 2% CH exhibited intermediate properties, while 1_50 and 1_60, with the lowest concentration of CH (1%), displayed a weaker structure. This is consistent with the findings of Sánchez-Cid et al. (2021) [26], who demonstrated that increasing the concentration of CH in hydrogels significantly enhances the rigidity and cohesion of the polymer network. On the other hand, as observed, oleogels 1_50 and 1_60, which share the same concentration of CH but differ in their O/W ratio, did not show significant differences (*p* < 0.05) in the rheological results. Therefore, it can be concluded that, in this case, the O/W ratio did not affect the final oleogel structure when the concentration of CH was fixed. This contrasts with previous findings in the literature, where the O/W ratio typically has a clear influence on the outcome [27,28]. For instance, Su et al. (2019) [29] reported a greater influence of the O/W ratio on rheology, but in systems with wider oil ranges (O/W ratio from 50:50 to 0:100) and lower structuring concentration (0.6% hydroxyethylcellulose). It should be noted that those studies employed different oils and different gelling agents derived from starch. These differences in formulation may explain the absence of the O/W effect in our study. Additionally, to the best of our knowledge, no previous studies have investigated an O/W variable using CH as a structuring agent acting through the Schiff base reaction. Therefore, the absence of the O/W effect can be explained by the relatively narrow O/W range studied and by the strong structuring effect of CH at the concentrations used (1–3%), which dominates the system’s behavior and masks the impact of the oil phase.

In general, both moduli increased with higher concentration of CH and lower oil phase proportions in the O/W ratio. In agreement with this, the following empirical models were obtained by fitting the experimental data obtained from experimental design G′ = 2.14·10^5^ + 1.29·10^5^·[CH] − 3.19·10^5^·[O/W]] − 3.21·10^5^·[CH]·[O/W] and G″ = 2.52·10^4^ + 1.77·10^4^·[CH]] − 3.25·10^4^·[O/W] − 3.24·10^4^·[CH]·[O/W]. These regression models illustrate how the concentration of CH and the O/W ratio, as well as their interaction, influence the elastic and viscous moduli of the oleogels. The results suggest that higher CH levels and lower O/W ratios enhance the oleogels’ rigidity and resistance. Therefore, the models serve as predictive tools for optimizing rheological properties and efficiently designing oleogels with tailored characteristics. The resulting response surface model for both moduli are depicted in Figure 2C,D. Supporting these regression models, strong positive correlations were found between G′ and G″ and the concentration of CH (r = 0.842 and r = 0.854; *p* < 0.01, respectively), and moderate negative correlations with the O/W ratio (r = −0.529 and r = −0.521; *p* < 0.05, respectively).

The frequency sweep results showed that G′ was consistently higher than G″, indicating a predominantly elastic behavior across all formulations. To further interpret these rheological patterns, the damping factor (tan δ = G″/G′) was analyzed, with values ranging from 0.085 to 0.129. Since tan δ values below 1 reflect elastic dominance, these findings confirm the formation of structured oleogel networks with solid-like properties [30].

### 3.3. Thermal Properties of Oleogels

**Figure 3:**
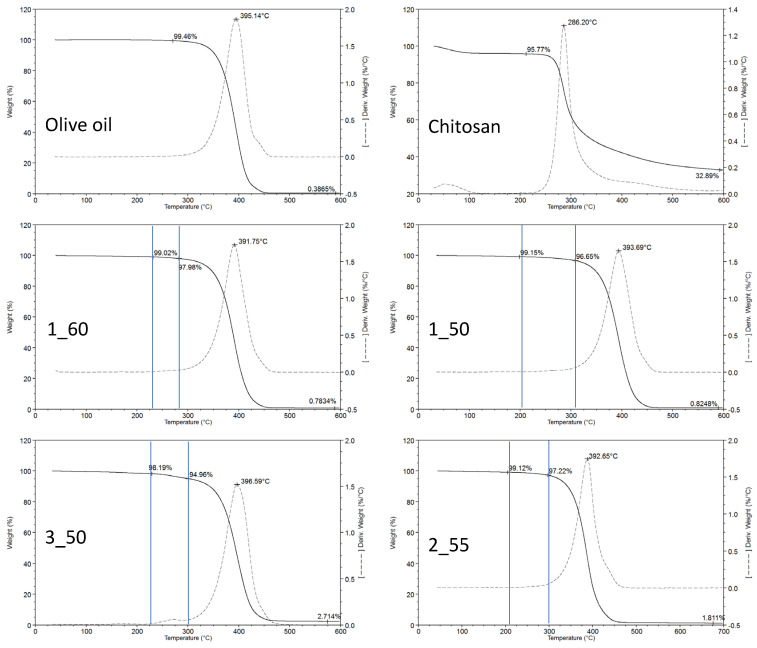
TGA curves of the olive oil oleogels elaborated with different chitosan concentrations and O/W ratio. Sample 1_60 (1% CH, O/W = 60); sample 1_50 (1% CH, O/W = 50); sample 3_50 (3% CH, O/W = 50); sample 2_55 (2% CH, O/W = 55). Vertical blue lines indicate the mass loss mainly influenced by the chitosan decomposition. Tmax values for chitosan are not represented, as they are not clearly defined due to the overlapping of the oil decomposition step.

Regarding the TGA of the individual pure components, it is clearly observed that CH provides a substantially greater amount of residual mass at 600 °C when compared to the oil constituent (Figure 3). Specifically, CH contributes a residue of 32.9%, whereas olive oil accounts for only 0.4%. These remaining masses are primarily associated with non-volatile fractions—including inorganic salts and mineral compounds—characteristic of the structural composition of CH and absent in the oil phase. Thermal decompositions were interpreted using the peaks observed in the first derivative thermogravimetric curves (DTG), which represent the temperature of the maximum decomposition rate for each constituent, herein referred to as Tmax, used to identify the decomposition steps of each constituent of the oleogel. For the pure substances, Tmax values determined were 395 °C and 286 °C for olive oil and CH, respectively. Both Tmax values are discernible in the thermograms of the analyzed oleogels, allowing for identification of the decomposition of each component within the formulation.

In the thermograms of the oleogel samples (Figure 3), the Tmax of chitosan appears to be consistently shifted to lower temperatures, averaging around 269 °C ± 3 °C. This shift may indicate alterations in the thermal stability of CH due to matrix interactions or formulation effects. Conversely, the Tmax of the oil remains essentially unchanged, observed at 392 °C ± 7 °C, suggesting that its thermal behavior is unaffected by the presence of other constituents. The overall mass loss percentages at each decomposition stage demonstrate a strong correlation with the relative concentrations of chitosan and oil in the respective formulations. Prior to these decomposition stages, no evidence of water evaporation was detected, implying that water was not retained within the oleogel matrix under the conditions examined. No significant decomposition was observed in any thermogram at a temperature lower than 200 °C, thereby permitting the use of these oleogels in low-temperature cooking processes.

Following complete thermal degradation, the final residue at 600 °C directly reflects the proportion of CH present in each oleogel. As demonstrated in the thermogram of pure CH, it serves as the principal contributor to this high-temperature residue due to its inherent composition that is rich in non-volatile materials. Consequently, the post-decomposition residual mass provides an indicator of CH loading within the oleogel and highlights its dominant role in determining the inorganic residue profile observed at elevated temperatures.

### 3.4. Oil-Binding Capacity and Textural Profile Analysis

Oil-binding capacity (*OBC*) is the ability of an oleogel to retain oil within its three-dimensional network. High *OBC* values (≥90%) are crucial to ensure effective oil entrapment and structural stability. All formulated oleogels achieved an *OBC* above 90%, except for those formulated with 1% CH and 60% of oil (Table 2). It should be noted that increasing the concentration of CH beyond 2% did not yield significant improvements in *OBC*, suggesting that further addition of the gelling agent is not necessary. Linear regression gave the model (*OBC* = 96.79 + 8.63·[CH] + 5.61·[CH]·[O/W]), showing that the *OBC* rises mainly with the concentration of CH while its interaction with the O/W ratio provides a smaller gain. Consistently, the *OBC* correlated positively with the concentration of CH (r = 0.744, *p* < 0.01) and negatively with oil content in the emulsion (r = −0.636, *p* < 0.01).

**Figure 4:**
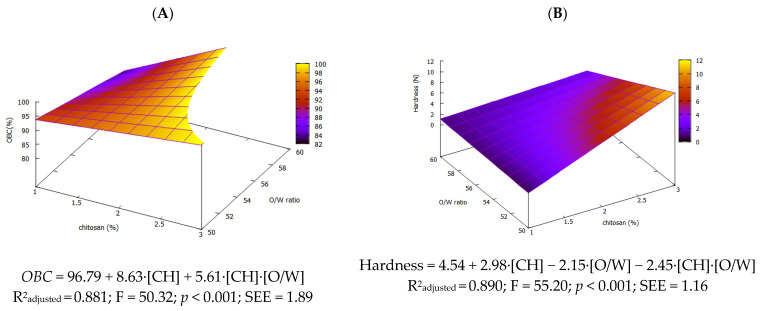
Response surface for *OBC* (**A**) and hardness (**B**) of olive oil oleogels.

The dominance of CH concentration is clear in the response surface plot (Figure 4A), highlighting its strong influence within the modeled region.

Similar behavior was reported by Farooq et al. (2023) [5] who observed an increase in *OBC* with higher chitosan concentrations working with oil bodies from camelia seeds. In our study, oleogels containing a concentration of CH above 2% exhibited *OBC* values exceeding 95%, indicating a robust molecular network and excellent oil retention capacity, even under stress conditions, as reported by Brito et al. (2022) [7]. These results align with the findings of Zhu et al. (2024) [11], who observed similarly high *OBC* values (>95%) in soybean oil oleogels formulated with chitosan and stabilized using 4-hydroxybenzaldehyde.

The textural properties of food products are critical, as they directly influence the final product and, consequently, the consumer’s sensory experience. The hardness values of the oleogels varied widely, ranging from 1.26 to 12.13 N, demonstrating the high versatility of CH, which enabled the development of products with diverse specifications suitable for various culinary applications. The hardness of the oleogels differed significantly (*p* < 0.05) between systems containing 1% and 3% chitosan (CH). However, for systems with 1% CH, the hardness did not vary with changes in the oil-to-water (O/W) ratio. Central point replicates (2 % CH) were highly reproducible and had intermediate hardness (Table 2).

As shown in the hardness response surface (Figure 4B), the model (hardness = 4.54 + 2.98·[CH] − 2.15·[O/W] − 2.45·[CH]·[O/W]) demonstrates that both factors and their interaction significantly influence hardness, with chitosan concentration exerting the strongest effect. Increasing the concentration of CH yields a denser, more extensive molecular network and thus higher hardness, consistent with the findings by Lama et al. (2024) [9]. In contrast, the impact of O/W ratio on hardness has received little attention. Our data show that increasing oil content during emulsion reduces hardness, likely due to a less compact molecular network as oil occupies more intramolecular space. Although adding more chitosan can partly compensate, this works only up to a point—e.g., oleogels with 3% CH failed at 60 % oil. As with other oleogels, a higher gelling agent level consistently increases hardness, as demonstrated by Yilmaz et al. (2021) [10] in virgin olive oil.

Cohesiveness values ranged from 21.84% to 29.39%, showing a slight tendency to increase as the concentration of CH and oil content decreased (i.e., in softer oleogels); meanwhile, springiness ranged from 0.296 to 0.659 mm. Despite the variation in the amount of CH, no significant differences (*p* > 0.05) were found in the mean values of cohesiveness or springiness, due to high standard deviation (Table 2). In more compact, cross-linked networks exhibiting higher hardness, the system resists deformation prior to rupture due to both increased molecular repulsion and network integrity. This enhances both cohesiveness and springiness along with hardness, as previously observed by Farooq et al. (2023) [5] in oleogels formulated with κ-carrageenan/chitosan. On the contrary, Brito et al. (2022) [7] reported an inverse relationship between springiness/cohesiveness and hardness in olive oil oleogels formulated with vanillin/chitosan. Both studies indicate that the type and amount of cross-linking agents determine the polymer network structure, significantly influencing the balance among hardness, springiness, and cohesiveness. Specifically, rigid polymer networks tend to exhibit increased hardness at the expense of springiness and cohesiveness, whereas dynamic networks allow these properties to coexist simultaneously. Adhesiveness (−1.18 to −0.32 N·s) decreased with chitosan concentration (r = −0.743, *p* < 0.01) and was strongly, inversely correlated with hardness (r = −0.872, *p* < 0.01). Only 3 % chitosan oleogels differed significantly (*p* < 0.05) from the rest.

Extensibility, inversely related to hardness, is one of the most desirable properties in fats mimetics [7]. Thus, our hardest gel (12.1 N) would suit meat products, whose matrix hardness ranges between 10 and 15 N, such as beef (14.84 N) or processed chicken products like nuggets (10.86 N) [31]. By contrast, the 1 % chitosan gels (1.25–1.86 N) match the range reported for butter substitutes (1.0–3.3 N) and could act as margarine replacements [32].

### 3.5. Relationship Among Rheological, Textural Properties, and OBC

Rheological results indicated that increasing CH concentration led to a more compact molecular structure, resulting in a more pronounced solid-like behavior. In agreement with this, a strong positive correlation was found between oleogel G′ and hardness (r = 0.953, *p* < 0.01), suggesting that both parameters are governed by similar structural characteristics, indicating that greater network strength contributes to higher resistance to deformation. This relationship has been demonstrated by Wijarnprecha et al. (2018) [33] who showed that increasing the concentration of rice bran wax in rice bran oil led to simultaneous increases in both elastic modulus and hardness, confirming that these parameters reflect the structuring ability of the gel network. Conversely, a negative correlation was observed between oleogel G and adhesiveness (r= −0.817, *p* < 0.01), suggesting that firmer gel networks reduce surface stickiness, likely due to restricted molecular mobility at the interface. Additionally, a moderate positive correlation was found between G′ and *OBC* (r = 0.491, *p* < 0.05). This relationship agrees with previous findings [11,34]. In line with this, Su et al. (2024) [35] reported that an increased degree of lipid unsaturation enhanced both the structural firmness and the oil retention capacity of oleogels. These results suggest that a more compact and stronger gel network, reflected by higher G′ values, improves the ability of oleogels to trap and retain oil effectively, thereby increasing their *OBC*.

### 3.6. Visual Appearance and Color Features

Color is a key physicochemical parameter in the characterization of oleogels, exerting a significant influence on consumer acceptance, particularly when evaluating alternatives to conventional fat. Additionally, during the reaction between CH and 4-hydroxybenzaldehyde to form Schiff bases, a characteristic color can be developed whose intensity may vary depending on the extent of the reaction. Color characteristics of the oleogels are shown in Table 3.

**Figure 5:**
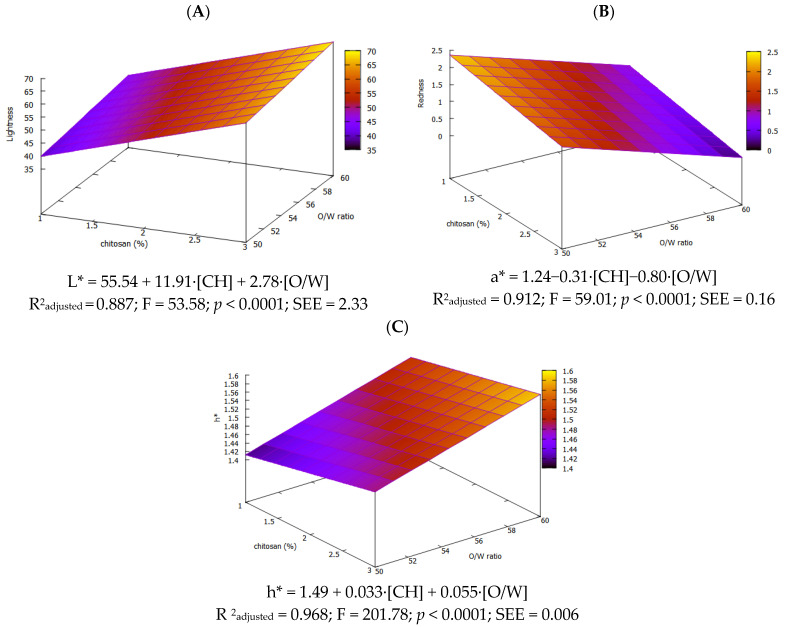
Response surface for L* (**A**), a* (**B**) and h* (**C**) of olive oil oleogels.

It was noticed that oleogels with [CH] ≥ 1% exhibited L* > 50, which according to Barragán-Martínez et al. (2022) [36], can be considered indicative of high lightness. Indeed, significant differences (*p* < 0.05) found by the [CH] factor are supported by the model for L* (L* = 55.54 + 11.91·[CH] + 2.78·[O/W]), where the coefficient for [CH] is more than 20 higher than that of O/W, and L* correlates strongly with CH concentration (r = 0.933, *p* < 0.01). A similar pattern has been reported for vanillin-linked olive gels [9]. Regarding a* and b*, both were positive with b* being far larger, giving a yellow cast. Based on the model equations (a* = 1.24−0.31·[CH]−0.80·[O/W] and b* = 18.52 + 2.85·[CH]), redness decreases as CH or oil rises (r = −0.866; *p* < 0.01 for O/W), whereas yellowness increases with CH (r = 0.763; *p* < 0.01). Chroma depends only on chitosan concentration (C * = 18.50 + 2.73·[CH]) and hue varies with both factors (h* = 1.49 + 0.033·[CH] + 0.055·[O/W]). Figure 5 illustrates the RSM for the three coordinates, which depends on two factors.

Overall, the color differences among all oleogels indicate that increasing the amount of CH raises the extent of Schiff-base cross-linking reaction, producing denser networks with distinct optical signatures, which is an effect also previously noted by other authors [7,35].

### 3.7. Oxidation Degree of Oleogels

**Figure 6:**
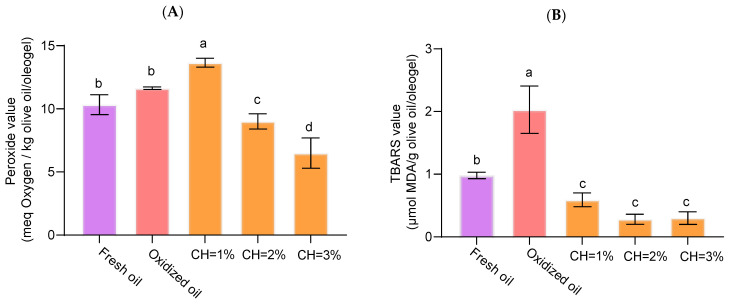
Primary (**A**) and secondary (**B**) oxidation in olive oleogels. Effect of chitosan concentration in comparison with fresh olive and oxidized olive oil in the same conditions (70°C for 3 h). Different letters denote statistically significant differences (α < 0.05) according to Duncan post hoc test.

Oil oxidation produced by the drying step was followed by primary and secondary oxidation and only for the concentration of CH, as O/W had no significant effect. PV ranged from 6.49 to 13.66 meq O_2_/kg for oleogels structured with 3% and 1% of chitosan, respectively (Figure 6A), below the maximum limit of 20 meq O_2_/kg of oil established by European regulations [37] for extra virgin olive oil. Significant differences (*p* < 0.05) were observed among the three concentrations of CH used, with intermediate PV for both fresh and oxidized olive oils. When the IP values of the oxidized oil were compared under identical conditions (70 °C, 3 h) with those of the oleogels, a reduction in IP inhibition was evident, with values of 22.52% and 44.19% for oleogels containing 2% and 3% CH, respectively. This progressive decline indicates that increasing chitosan concentration enhances the protective effect against primary oxidation. Therefore, [CH] ≥ 2 % exerts a clear protective effect, probably by forming a dense, positively charged shell that restricts oxygen diffusion [38]. Additionally, CH molecules covering the olive oil can function as a coating layer that scavenges oxidants at the interface thereby potentially slowing the formation of lipid oxidation [5]. Moreover, the para-substituted phenolic ring of 4-hydroxybenzaldehyde likely scavenges radicals, further inhibiting lipid oxidation.

The TBARS values ranged from 0.3 to 2.0 μmol MDA/g olive oil/oleogel. Significant differences (*p* < 0.05) between olive oil and oleogels were found (Figure 6B). Despite there being no significant differences (*p* > 0.05) regardless of chitosan dose, secondary oxidation significantly decreased (*p* < 0.05), indicating greater oxidative stability in the tested oleogels. In the case of secondary oxidation, the reduction in inhibition relative to the oxidized control was substantially greater, reaching 86.20% and 85.22% for 2% and 3% chitosan, respectively. These findings suggest that increasing chitosan concentration beyond 2% does not confer additional advantages in terms of secondary oxidation. These findings are consistent with those reported by Farooq et al. (2023) [5] who reported a decrease in TBARS in oleogels formed with CH concentration and vanillin. This suggests that chitosan plays an active role against non-structured oils in concordance with PV results. Indeed, a negative correlation between the concentration of CH and PV (r= −0.733, *p* < 0.01) was found.

### 3.8. In Vitro Digestibility of Oleogels

**Figure 7:**
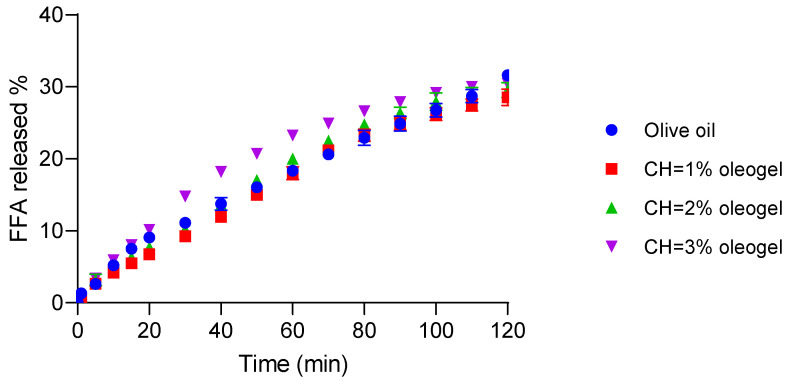
Digestibility of olive oil and oleogels structured with chitosan. Effect of chitosan concentration on the total free fatty acids.

The results of digestibility kinetics are depicted in Figure 7. At the final point (120 min), the olive oil control exhibited the highest digestibility (31.62 ± 0.12%), followed by oleogels of [CH] = 3% with 30.43 ± 0.56 which was not significantly different (*p* > 0.05) from the control. The other oleogels showed slightly lower digestibility values (29.55 ± 1.04 and 28.54 ± 1.14 for 2% and 1% [CH], respectively). There were significant differences (*p* < 0.05) between olive oil and 3% CH-oleogels, as well as between 3% and 1% CH-oleogels.

Overall, these results indicate that oleogels tend to undergo slightly slower digestion compared to pure olive oil, likely due to the structuring effect of CH forming a molecular network that restricts enzymatic access to the oil phase. However, the oleogel with the highest 3% CH and rigidity demonstrated digestibility comparable to the olive oil. This suggests that despite the initial barrier posed by the gel network, its increased rigidity combined with brittleness facilitates structural breakdown during digestion, promoting enhanced pancreatic lipase penetration and FFA release. These findings are consistent with Acevedo-Fani and Singh (2022) [39] who emphasized that the balance between rigidity and fragility in structured lipid systems is critical for modulating lipid digestibility. Similarly, other authors reported that CH and vanillin-based oleogels with compact but fracturable structures allow efficient digestion through the progressive exposure of the encapsulated oil [5].

In support of a mechanistic link between gel fracture, rheological behavior, and lipase access, Cofrades et al. (2024) [40] reported that gelled emulsions exhibited significantly higher lipid digestibility and a larger absorbable fraction (i.e., FFA + monoacylglycerol) than oleogels (~51%), which behaved similarly to bulk oil (~48.9%), showing lower digestibility. This supports the notion that the fracture or disintegration of the gel matrix in gelled emulsions increases interfacial area, enhances lipase access, and thus accelerates hydrolysis. In line with this, Luo et al. (2021) [41] demonstrated that whey protein emulsion gels with lower mechanical strength (“soft gels”) disintegrated more quickly during gastric digestion and released oil droplets that began to coalesce as early as 60 min, whereas “hard gels” remained intact longer and resisted breakdown. Overall, these findings illustrate two complementary mechanisms: first, the rate and extent of physical gel breakdown—whether in emulsion gels or protein gels—determine the generation of fragments or droplets with increased interfacial area; second, there is a quantitative relationship between the degree of lipolysis and the efficiency of bioactive-compound release. Together, these observations provide a mechanistic rationale for why tuning gel rigidity (structural integrity) and brittleness (fragmentation under stress) can enhance (for gelled emulsions) or maintain (for oleogels) lipid digestibility relative to bulk oil.

## 4. Conclusions

In this study, structured olive oil oleogels using chitosan were successfully developed by varying chitosan concentration and oil-in-water ratio. However, a technical limitation was encountered at 3% chitosan and a 60/40 oil-in-water ratio, where oleogel formation failed due to solubility and viscosity issues. Although chitosan concentration was the main factor affecting oleogel properties, the O/W ratio had a significant impact on emulsion stability—a critical factor for successful gel formation.

The rheological analysis showed that all successful formulations formed elastic gel networks characterized by a storage modulus consistently higher than the loss modulus and low damping factors, confirming the predominance of elastic behavior typical of structured gels. Increasing chitosan concentration enhanced the stiffness and structural integrity of the oleogels, as evidenced by higher viscoelastic moduli and increased brittleness in the most cross-linked systems. Additionally thermal analysis revealed strong resistance to thermal degradation, indicating its suitability for processing.

On the other hand, increasing the chitosan concentration to values higher than 2% did not significantly improve the oil-binding capacity, suggesting that further addition would be unnecessary and also counterproductive because the high gel hardness would create an inadequate oleogel texture that is ineffective as a fat replacement food component. This study demonstrated that oleogels with varying hardness and adhesiveness can be obtained, enabling their application in the design of different products such as mayonnaise, margarine, or other higher-hardness formulations like processed meat products.

The results support the hypothesis that chitosan acts as a physical barrier against oxidation, which could extend the shelf-life of oleogel-based products, particularly for meat applications. Oxidation during drying was low. Combined with controlled digestibility that improves nutritional value, this supports the use of chitosan–olive oil oleogels as stable, functional replacements for conventional fats in meat products.

## Figures and Tables

**Table 1:** Central composite design without axial points for each experimental system with coded and decoded independent variables (CH concentration and O/W ratio).

System	ch	o/w	CH (%)	O/W
1	−1	−1	1	50
2	+1	−1	3	50
3	−1	+1	1	60
4	+1	+1	3	60
5	0	0	2	55
6	0	0	2	55
7	0	0	2	55
8	0	0	2	55

CH = chitosan concentration; O/W = oil in water ratio.

**Table 2:** *OBC* and textural profile analysis of the olive oleogels for each experimental system (mean ± SD; *n* = 3).

System	*OBC* (%)	Hardness (N)	Cohesiveness (%)	Springiness (mm)	Adhesiveness (N·s)
1	93.41 ± 1.97 ^c^	1.25 ± 0.06 ^d^	25.98 ± 4.45	0.34 ± 0.03	−0.29 ± 0.10 ^a^
2	99.45 ± 0.94 ^a^	12.12 ± 2.49 ^a^	21.85 ± 2.75	0.39 ± 0.18	−1.18 ± 0.46 ^b^
3	82.92 ± 2.23 ^d^	1.86 ± 0.29 ^cd^	29.38 ± 3.02	0.45 ± 0.02	−0.35 ± 0.08 ^a^
5	94.55 ± 2.02 ^bc^	3.67 ± 0.55 ^bc^	24.44 ± 6.13	0.29 ± 0.07	−0.46 ± 0.05 ^a^
6	97.32 ± 1.71 ^ab^	4.82 ± 1.23 ^b^	22.36 ± 2.89	0.65 ± 0.08	−0.55 ± 0.19 ^a^
7	98.51 ± 0.19 ^a^	5.13 ± 1.25 ^b^	24.30 ± 2.38	0.59 ± 0.26	−0.34 ± 0.28 ^a^
8	96.82 ± 1.02 ^ab^	4.58 ± 0.25 ^b^	23.79 ± 2.96	0.61 ± 0.12	−0.52 ± 0.14 ^a^

Different letters in the same column indicate significant differences (α < 0.05) according to Duncan’s test.

**Table 3:** Color chromatic parameters of the olive oleogels for each experimental system (mean ± SD; *n* = 3).

System	L*	a*	b*	C*	H*	Real Color
1	43.05 ± 1.83 ^c^	2.41 ± 0.26 ^a^	14.64 ± 0.93 ^c^	14.83 ± 0.46 ^c^	1.40 ± 0.005 ^d^	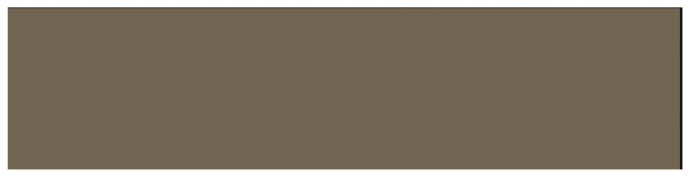
2	62.46 ± 1.16 ^a^	1.67 ± 0.08 ^b^	20.20 ± 0.73 ^a^	20.26 ± 0.73 ^a^	1.48 ± 0.005 ^c^	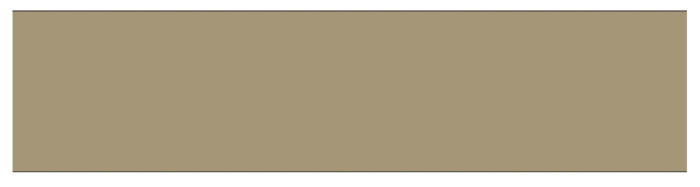
3	44.22 ± 6.11 ^c^	0.70 ± 0.27 ^d^	16.68 ± 2.94 ^bc^	16.69 ± 2.95 ^bc^	1.52 ± 0.01 ^a^	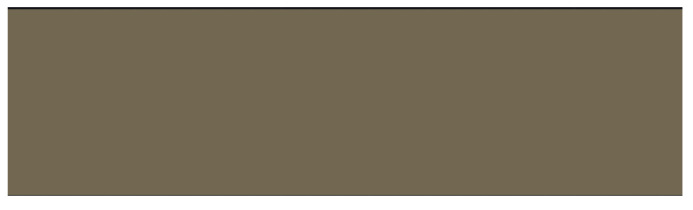
5	55.21 ± 1.46 ^b^	1.10 ± 0.10 ^c^	17.79 ± 1.15 ^ab^	17.82 ± 1.15 ^ab^	1.50 ± 0.005 ^b^	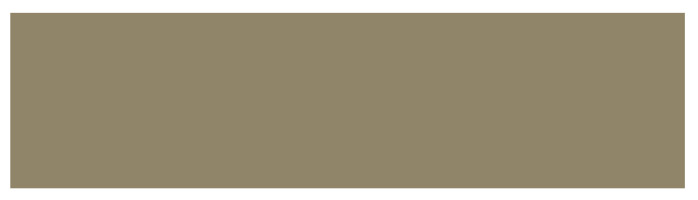
6	55.31 ± 0.86 ^b^	1.30 ± 0.92 ^c^	19.10 ± 0.10 ^ab^	19.14 ± 0.19 ^ab^	1.49 ± 0.005 ^b^	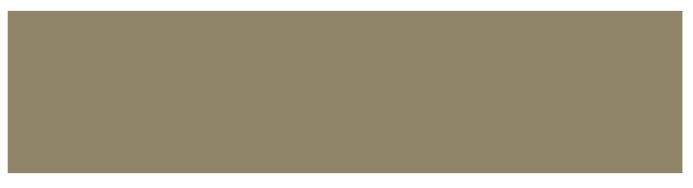
7	56.10 ± 0.48 ^b^	1.32 ± 0.12 ^c^	18.48 ± 0.33 ^ab^	18.52 ± 0.34 ^ab^	1.49 ± 0.005 ^b^	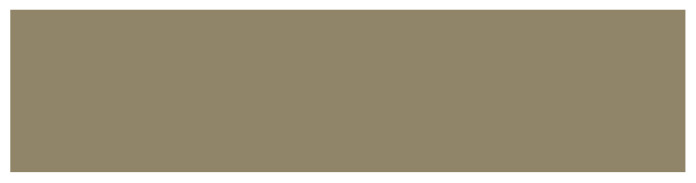 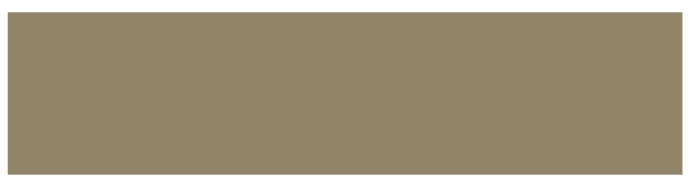
8	55.54 ± 0.51 ^b^	1.23 ± 0.09 ^c^	18.69 ± 0.78 ^ab^	18.73 ± 0.51 ^ab^	1.50 ± 0.001 ^b^	
Real Oleogels		1_50 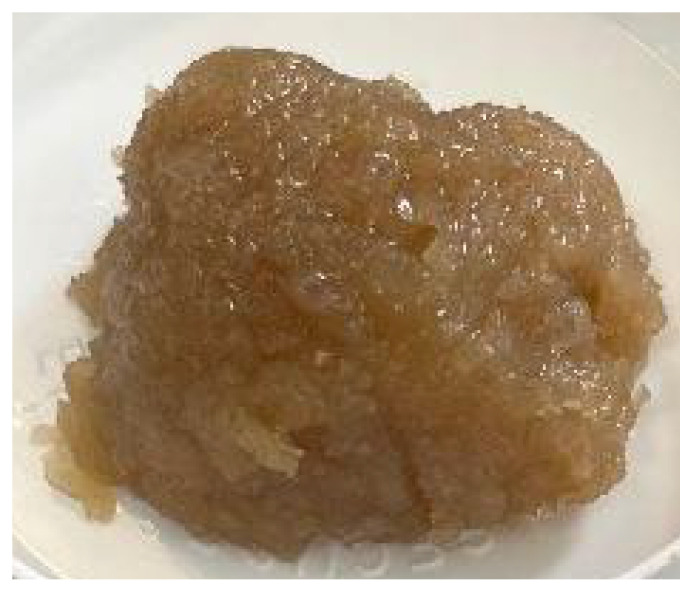	3_50 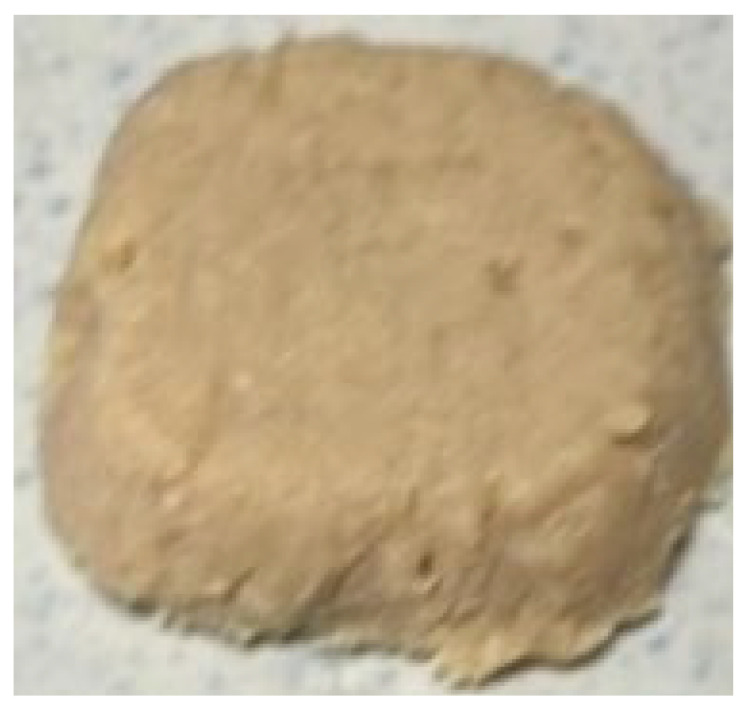	1_60 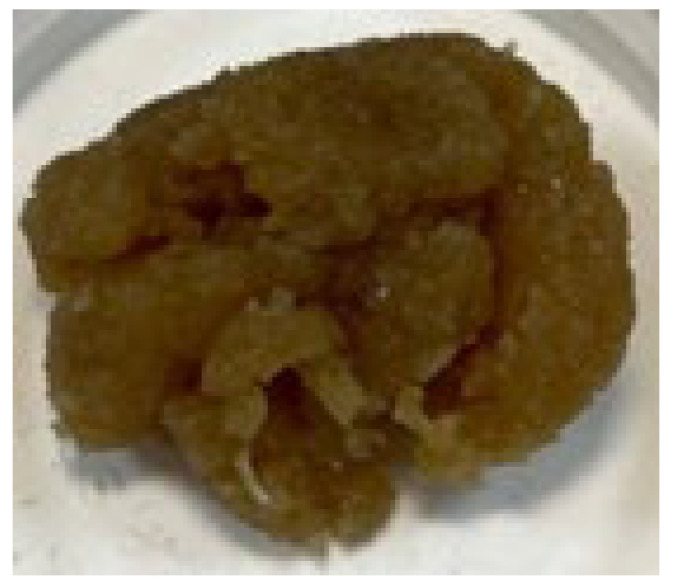	2_55 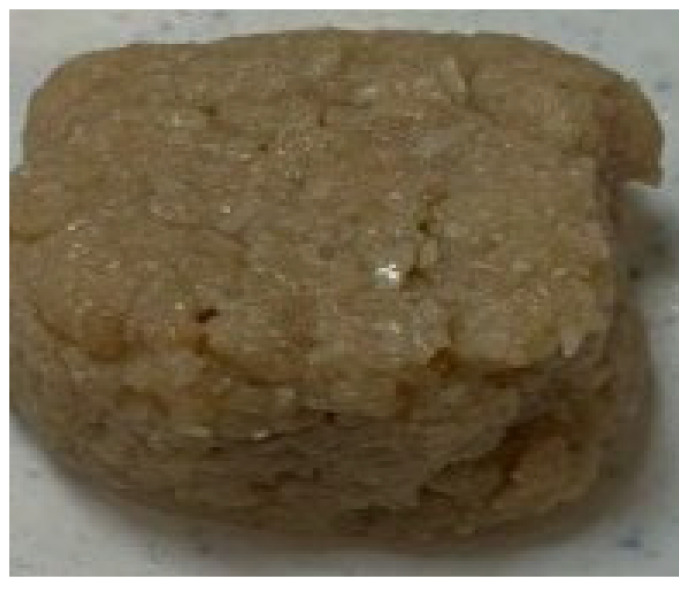	

Different letters in the same column indicate significant differences (α < 0.05) according to Duncan’s test.

## Data Availability

The original contributions presented in the study are included in the article, further inquiries can be directed to the corresponding author.
